# Community Knowledge, Attitude, and Practices Regarding Mosquitoes and Mosquito-Borne Viral Diseases in Kinshasa, Democratic Republic of the Congo

**DOI:** 10.3390/epidemiologia4010001

**Published:** 2022-12-30

**Authors:** Kennedy M. Mbanzulu, Leonard E. G. Mboera, Roger Wumba, Josué K. Zanga, Flory K. Luzolo, Gerald Misinzo, Sharadhuli I. Kimera

**Affiliations:** 1 SACIDS Africa Centre of Excellence for Infectious Diseases of Humans and Animals in Eastern and Southern Africa, Sokoine University of Agriculture, Chuo Kikuu, Morogoro P.O. Box 3297, Tanzania; 2 Department of Tropical Medicine, Infectious and Parasitic Diseases, University of Kinshasa, Kinshasa P.O. Box 747, Democratic Republic of the Congo; 3 Department of Veterinary Microbiology, Parasitology and Biotechnology, Sokoine University of Agriculture, Chuo Kikuu, Morogoro P.O. Box 3019, Tanzania; 4 Department of Veterinary Medicine and Public Health, Sokoine University of Agriculture, Chuo Kikuu, Morogoro P.O. Box 3021, Tanzania

**Keywords:** knowledge, attitude, practices, mosquitoes, mosquito-borne viruses, Democratic Republic of the Congo

## Abstract

Background and Objectives: Mosquito-borne viral diseases (MBVDs) create a dramatic health situation worldwide. There is a need to improve the understanding of factors to be addressed in intervention programmes. This study explored community knowledge, attitudes, and practices (KAP) regarding MBVD in Kinshasa. Materials and Methods: A cross-sectional survey was carried out between January and April 2019. The socio-demographic and KAP data collected through a questionnaire were analysed using Epi Info 7. Results: The study included 1464 male and female respondents aged from 18 to 70 years old. Open garbage cans and outdoor water storage units were found in 61.2% and 33.4% of respondent residences, respectively. Polluted water bodies (80.3%) were the most mentioned as mosquito breeding places. Among 86.6% of the respondents that had heard about yellow fever, 12% knew that it is an MBVD. The majority of respondents (72.5%) were perceived to be at risk of contracting MBVD. Environment sanitation (58%) and insecticide use (25%) were among the measures implemented to control mosquitoes. The greater overall knowledge score and attitude were not associated with good practice. Conclusion: The residents of Kinshasa had limited knowledge of MBVD. Raising awareness and educational sessions are essential in empowering the community regarding the correct attitudes and practices to effectively manage the risk posed by MBVD.

## 1. Introduction

Mosquitoes transmit different pathogens that affect human and animal health and negatively impact food security and socio-economic wellbeing [1,2,3]. In addition to malaria and lymphatic filariasis, mosquitoes are also vectors of several viral diseases. The most important mosquito-borne viral diseases (MBVDs) include yellow fever, Zika, dengue, chikungunya, Rift Valley fever, and West Nile [4]. Transmission of MBVD to humans and animals includes multifaceted processes which are influenced by mosquito and viral genetic, environmental, socio-demographic, and anthropological factors [5,6].

For effective interventions, in addition to knowledge of biomedical aspects of the diseases, information on socio-anthropological aspects is equally important. It is critical to explore different local socio-cultural and demographic driving factors of MBVD in order to design appropriate interventions. In the current context of increasing insecticide resistance, limited vaccine options, and lack of curative resources, an integrated approach based on community and individual participation are critical in the effective prevention and control of MBVDs.

There is limited information on community knowledge, attitudes, and practices (KAP) on MBVD in Sub-Saharan Africa [7]. Studies outside Africa have shown that KAP on MBVD vary widely across populations and countries [8,9,10,11,12,13]. Inadequate knowledge is a significant barrier to appropriately empowering local communities and individual interventions against MBVD. Lack of or inadequate community knowledge is likely to be an obstacle in adopting specific prevention and control measures against some specific mosquito species and MBVD [11].

Indeed, mosquito species express different biting behaviour and breeding preferences. Generally, populations are often discussing mosquitoes in a global way and do not differentiate between mosquito species. Such conceptualisation could pose an obstacle to the adoption of specific prevention or control measures for some specific mosquito species and MBVDs [13]. Contrary to Anopheles, the main vector of malaria and o’nyong’nyong virus (ONNV), which present nocturnal activities, Aedes aegypti, the main vector of yellow fever virus (YFV), Zika virus (ZIKV), dengue virus (DENV), and chikungunya virus (CHIKV), present a diurnal activity and preferentially breed in domestic containers and bite in peridomestic locations. Culex pipiens complex, a major vector of WNV, tend to breed in polluted water bodies containing organic matter [14,15,16,17,18]. Considering such details in educating the population could raise their awareness of the vector, the viruses, and adapted control and prevention measures.

The Democratic Republic of the Congo (DRC) experiences both tropical and equatorial climates with long rains. The climatic and ecological conditions are optimal for almost all major MBVDs of public health importance. MBVDs are becoming common and a serious public health problem in the DRC [19]. The country is known to be at high risk of YFV transmission, morbidity, and mortality [20]. More than 400 people died during the yellow fever outbreaks of 2016–2017 [6,21]. Kinshasa, the capital city, has experienced four chikungunya outbreaks during the past two decades [22,23,24]. Recently, reports of dengue occurrence have increased [23,25,26,27], the presence of Zika virus (ZIKV) has been documented [27], and the overall seroprevalence of Rift Valley fever virus (RVFV) has increased [28]. To date, Aedes albopictus has been reported in Kinshasa [29]. These threats of MBVD are not only to the local population of the DRC but also to visitors. For instance, the majority of chikungunya virus infections in Belgium between 2007 and 2012 were imported from the DRC [30] and, recently, Japanese and Italian travelers returning from the DRC were diagnosed with DENV [31,32]. The evidence of circulating West Nile virus (WNV) in dogs, horses, and mosquitoes has been documented in Kinshasa [33,34,35]. In the context of inadequate resources for control, there is an immediate need to increase community awareness of MBVD in the DRC. This study was therefore carried out to determine community knowledge, attitudes, and practices as regards mosquitoes and MBVDs in Kinshasa, DRC.

## 2. Materials and Methods

### 2.1. Study Area and Design

In this cross-section study, a questionnaire survey was conducted in four districts of Kinshasa between January and April 2019. Kinshasa has 24 communes (municipalities) grouped into four districts and each commune is divided into neighbourhoods. It has an estimated human population of 11,855,000 [36]. A multistage sampling technique was carried out to select study participants. At the first level, three municipalities from each district of Kinshasa were chosen. At the second level, two neighbourhoods were selected to guarantee a good coverage of the geographical, demographic, and socio-economic profiles of the population. The head of the household or a representative was systematically selected from neighbourhoods. The participants included in this study have complied with the following criteria: (i) aged 18 years old or above, (ii) living in the selected neighbourhoods, and (iii) freely consent to participate in the study and being present during the interview.

A questionnaire was developed in English, translated into French, and administered by a face-to-face interview in either Lingala or French depending on the language proficiency of the respondent. The questionnaire contained both closed and open questions with the possibility for the respondent to provide more than one answer. The information sought was related to socio-demographic characteristics of respondents, knowledge about mosquitoes (breeding places, activities, behaviour, vector role, control measures, symptoms), attitudes and practices towards mosquitoes, and MBVD. The attitude questions were focused on the perception of mosquitoes’ impact on daily life and the consciousness about responsibility for individual and community protection against mosquitoes and MBVD. The practice questions captured the information about measures undertaken to reduce or avoid mosquito bites, and mosquito abundance on the property (environmental hygiene, use of chemical and physical means). To gain more insight and accuracy on the respondent’s practice, additional data were captured by inspection of their residential places and their surroundings (presence of vegetation, stagnant water collection, uncovered storage water unit set outdoors, any potential artificial or natural water container, opened garbage can).

### 2.2. Data Analysis

The data were entered into a Microsoft Excel spreadsheet and statistical analysis was performed using Epi Info Software Version 7 (CDC, Atlanta, GA, USA). A summary of the statistics is presented as frequencies and proportions in tables. Each correct response to the knowledge, attitude, and practice questions was scored on a scale of one to five while an incorrect response was assigned zero points. The sum of each knowledge component (breeding site, times of activity, vector role of mosquito in spreading viruses, arbovirus known or heard of before the survey, prevention, and control measures) was used to determine the overall knowledge score about MBVD by calculating the mean. The attitude questions sought information on how they perceived the impact of mosquitoes on daily life and their responsibility in prevention and protection. Awareness of health risks posed by mosquitoes and personal responsibilities at the household and community level for the prevention and protection of themselves, their households, and the community against mosquitoes and MBVD was considered to be a positive attitude. The preventive measures that have been undertaken and the information on the description of the immediate residential environment of the respondent were included in the determination of the overall practice score. Low scores were values less than the mean and high scores were values equal to or greater than the mean. The scoring procedures for each KAP component are provided in the Supplementary Materials. The total possible points were 30, 15, and 12 for overall knowledge, attitude, and practice, respectively.

The chi-square test was used to identify associated factors of the KAP scores by calculating the odds ratios (OR) and the 95% confidence interval (CI). *p*-values less than 0.05 were considered statistically significant. The socio-demographic characteristics (age, sex, education, occupation, marital status, religion) were considered independent variables.

## 3. Results

### 3.1. Socio-Demographic and Environmental Characteristics

Of 1464 respondents included in the study, 60.7% were females, 52.5% were above 33 years of age, and 43.2% had a higher level of education. About half of the respondents were married (Table 1), approximately half (47.8%) owned a house. The majority of the houses (61.2%) had open garbage cans and 38.7% had vegetation in their surroundings. One-third (33.4%) of the houses had water storage units set outdoors, 25.1% had stagnant water collections, and 22.5% had potential artificial or natural water containers outdoors (tyres, flower pots, small cans, boxes, coconut shells, plastic plates). Domestic animals were found in around one-third of the respondents’ homes. Only 36.4% of houses had insect screen windows. More details of the surveyed households are provided in Table 2.

### 3.2. Knowledge

The majority of respondents stated that stagnant and draining polluted water (80.3%) and garbage (35%) were the major mosquito breeding sites. As regards mosquito biting time, 39% considered mosquitoes to bite during the night, 31% during sundown, and 30.5% any time of the day. The majority of respondents mentioned environmental measures such as cleaning and removal of garbage (64.2%), draining of standing water (24.8%), and proper disposal of empty containers (10.1%) as the most effective mosquito control measures. Other measures are detailed in Table 3. Yellow fever was the most frequent (86.6%) MBVD that respondents had heard of before our study. Others included chikungunya (13.9%), Zika (7.5%), and dengue (3.7%). Only a few respondents knew that YFV (12.2%), CHIKV (5.4%), ZIKV (1.8%), DENV (1.5%), and RVFV (0.6%) were transmitted by mosquitoes. Almost all respondents (97.2%) identified malaria as a disease that is spread by mosquitoes. Regarding knowledge of the role of the mosquito in spreading zoonoses, only 23.7% (348/1464) were aware that mosquitoes can transmit pathogens to animals or exchange pathogens between animals and humans. Of these, 348 respondents (39.0%) were unable to mention any zoonosis (Table 4).

The majority (70.1%) of respondents who knew about any MBVD stated fever as the most common symptom, followed by headache (52.4%), general body pain (21.2%), and joint pain (18.7%). Only a few respondents mentioned jaundice (9.9%), back pain (4.2%), haemorrhage (2.2%), skin rashes (1.8%), and others (5.7%).

A total of 1346 (91.9%) participants had a low overall score of knowledge related to mosquitoes and MBVD (Table 5). Knowledge scores related to mosquito breeding places were significantly associated with the respondent’s age, marital status, educational level, and sex. Respondents above 33 years of age (OR: 2.4; 95%CI: 1.4–4.2; *p* = 0.0002), married (OR: 2.2; 95%CI: 1.3–3.7; *p* = 0.0016), and having higher educational level (OR: 2.0; 95%CI: 1.2–3.2; *p* = 0.002) had higher knowledge on mosquito breeding places. Compared to males, female respondents had low knowledge scores regarding mosquito breeding places (OR: 0.3; 95%CI: 0.2–0.6; *p* = 0.0001) and times of mosquito biting activity (OR: 0.8; 95%CI: 0.6–1; *p* = 0.03). The non-Christians (OR: 1.3; 95%CI: 0.9–1.7; *p* = 0.03) had higher scores than Christians about times of mosquito activity. The age, sex, occupation, religion, marital status, and level of education of respondents were not significantly associated with the knowledge of the role of the mosquitoes in transmission of zoonosis and arboviruses, arboviral disease, prevention and control measures, as well as the overall knowledge (Table 6).

### 3.3. Attitudes and Perceptions

Approximately three-quarters (72.5%) of the respondents perceived the impact of mosquitoes on their daily life. Most (60.7%) of them reported being bitten by mosquitoes outdoor in their home places, fewer at recreational places or workplaces, and half responded that they were bitten indoors. In all, 44.6% of respondents were regularly bitten and 31.2% reported sometimes. Overall, 90% of participants were bitten during dark hours (sundown 36%, night 53%); fewer reported being bitten during the day (7.0%). According to their experience, the respondents associated the mosquito abundance in residential places with the presence of drainage and blocked draining water channels (21%), garbage (17.7%), farming activities (14%), house/road construction (10%), and animal rearing (7.7%). The most familiar sources for searching for information about MBVDs were health professionals/hospitals (40.2%) and their relatives or family members (26.1%), radio or television (25.3%), and schools (17.7%), and the others reported in Table 7.

Most respondents (72.9%) perceived that they were responsible for the prevention and protection of themselves and their households against mosquitoes and MBVD. However, only 37.3% were aware of their responsibilities at the community level. They perceived that mosquitoes and MBVD control and prevention should be the responsibility of the health authorities and national government (Table 8).

About 80% appeared to observe the correct attitude towards MBVDs (Table 5). The overall attitude scores were significantly associated with the respondent’s age and occupation. Respondents aged over 33 years (OR: 0.8; 95%CI: 0.6–1.0; *p* = 0.02) had lower attitude scores compared to those aged 18 to 33 years. Considered together, students and medical personnel (OR: 0.002; 95%CI: 1.1–1.9; *p* = 0.002) had a correct attitude towards mosquitoes and MBVDs. The sex, religion, marital status, education, and overall knowledge were not significantly associated with the respondents’ attitudes (Table 9).

### 3.4. Practices Regarding Vector Control

Slightly more than a half (58.6%) of the respondents reported cleaning the environment, one-quarter used insecticides, and another one-quarter reported emptying garbage containers and emptying flower pots (11%) as the measures undertaken to reduce mosquito abundance around their homes. The draining of standing water was mentioned by 16.3% of respondents and garbage cleaning by 11.3%. Covering of water sources or drinking water and/or storage containers was stated by only 10.4% of respondents.

As regards measures undertaken to reduce or avoid mosquito bites, a large proportion of the respondents (79%) stated the use of mosquito nets, fumigation and spraying of insecticide (15.8%), mosquito screens on windows (13%), use of fans (10%), wearing long clothes (0.3%), and praying to God (1%). High proportions of residents (67.7%) confirmed that they did not have any challenge in taking action to prevent or control mosquitoes. Challenges in mosquito control and prevention included lack of money and other resources (42.9%), limited access to necessary items (19.3%), not having time (19%), and disbelief in the effectiveness of these preventive measures (12.8%). Although 87.4% of the respondents had at least one mosquito net, only 67% confirmed to have slept under a mosquito net during the previous night. The source of the mosquito nets included a national mass distribution campaign (68.8%), healthcare facilities (15%), and procurement from shops/markets (18.8%). Almost 45% of these mosquito nets had holes (Table 10). The overall practice score was lower among 85.7% of participants. The age, sex, occupation, marital status, and education of participants were not significantly associated with their practices. Believers other than Christians (OR: 0.5; 95%CI: 0.3–0.8; *p* = 0.003) had lower practices compared to the latter. A high overall knowledge (OR: 1.4; 95%CI: 0.8–2.3; *p* = 0.1) and attitude (OR: 1.22; 95%CI: 0.9–1.6; *p* =0.1) were not significantly associated with the respondents’ good practice (Table 11).

## 4. Discussion

The present study explored the level of community KAP concerning mosquitoes and MBVD in Kinshasa, DRC. The majority of respondents reported being frequently bitten by mosquitoes either outdoors or indoors and most stated that mosquito activities were more intense from sundown to night. Only a few participants knew about the daily activity of mosquitoes. A high proportion of study participants felt more concerned about health problems that are brought by mosquitoes. The observation of the residential environment of the respondents allowed taking inventory of the diverse types of human-made and natural containers that could serve as mosquito breeding places. This observation was in contrast with a good level of general knowledge about environmental preventive measures noted among the majority of respondents and what they confirmed as their usual practices towards control and prevention of mosquitoes. This confirms that often people do not properly understand the meaning of the concept of environmental management [13].

The majority of respondents emphasised environmental cleaning although a high percentage of uncovered garbage cans, vegetation, stagnant water collections, and abandoned domestic containers were present in residential places. In addition, probably due to inadequate water supply in some homes, people have set up different water storage units outdoors being unaware of a possible invasion of Aedes mosquitoes [37]. This confirms that the common Aedes breeding habitats are not well known by the majority of the respondents [38]. The most common mosquito breeding places known by the study population were polluted water bodies. Garbage places were perceived as the main drivers leading to mosquito abundance. This was in line with studies carried out in India [39,40].

The mechanical automobile activities that take place in the city might also contribute to mosquito abundance. Similar reports from Tanzania have indicated that tyres are among the most prolific breeding sites for Aedes mosquitoes [41]. Agriculture and construction of roads and houses were also reported among the activities leading to mosquito abundance in Kinshasa. These observations were consistent with findings reported from Kenya, Tanzania, Sudan, France, and the French Antilles [1,42,43,44]. Therefore, the messages for MBVD prevention should raise awareness among the stakeholders engaged in the design, materials, and all human resources such as architects, landscapers, construction professionals, distributors, and installers [44].

Nevertheless, the majority of respondents in the current study were unaware of the vector role of mosquitoes in spreading pathogens to animals and their involvement. Although the majority of study participants had heard of an Aedes-transmitted virus such as yellow fever, chikungunya, Zika, and dengue, the majority of them did not know that these viruses are transmitted to humans by mosquitoes. The Democratic Republic of the Congo has experienced four chikungunya and four yellow fever outbreaks during the past two decades [22,23,24,45,46,47,48]. This could be one of the reasons why the majority of the respondents were aware of these diseases.

The lack of knowledge on the role of mosquitoes in spreading viruses to both humans and animals could explain some contradictory attitudes, behaviours, and practices noted among study participants. Similar observations have been reported in Jamaica, where the population had poor knowledge of MBVD and poor prevention practices [49]. On the contrary, in Belize, more than 85% of the respondents confirmed that DENV, ZIKV, CHIKV, and YVFV are viruses transmitted by mosquitoes and that communities were regularly draining standing water or using insecticides to control mosquitoes [2]. Similar observations have been reported in Colombia, the USA, and China where the majority of the population was positively involved in source reduction preventive practices [12,50,51].

The appropriate knowledge of MBVD can empower individuals to make some effort to prevent or control MBVD in their properties instead of waiting for government intervention. Poor knowledge of MBVD has also been reported for RVF in Kenya, Tanzania, and Sudan [1,42,43]. The lack of knowledge is driving MBVD into new areas and leads to loss of life and economic losses [1,43]. The high level of dirt, multiple fortuitous markets, high demographic pressure, and inadequate urbanisation of the Kinshasa metropolitan area are suitable conditions to support the Culex mosquito, the main vector of WNV and RVFV [52,53,54]. In the DRC, currently, RVFV activities are increasing [28] and evidence of WNV in domestic dogs and horses has been documented from Kinshasa [34,35]. Regarding the number of households rearing either domestic or livestock animals in this study area, there is also an urgent need to raise awareness of the population about the role of the mosquito in spreading zoonosis.

Participants in the current study were less aware of how their involvement in the local population can boost the control of mosquitoes and MBVD in their community. The study participants thought that their duty was only for self-protection and their households but not for local community mosquito prevention and control. Similar observations have been reported in a study in Western Australia [55]. These positive attitudes of trusting in government action offer an opportunity for decision makers and health actors to maximise their educational activities in this community and to get closer to the population through its local structures. Even practically, the respondents did not perceive the responsibility of the local community and their role as a source of information. The population must perceive that control of mosquito-borne diseases does not only have to rely on individual or household protection but also protection at the community level. Strengthening cooperation between neighbouring households can also serve as an information channel to improve the knowledge levels of this study population. The financial limitation was mentioned as the main hindrance in taking action against mosquitoes for the majority of the study population. This could be the reason that the majority of study participants would resort less to control measures that incur expenditures. Once the health risk is perceived as a real threat and priority, the population can transfer their knowledge into action [13]. However, embracing protective behaviours is a multifactorial procedure influenced by socio-economic and cognitive factors [56]. In general, household expenditure on protective measures using chemicals is high [2,3]. So, in limited resource settings, it is better to emphasise environmental measures which are more accommodated, simple to implement, and very effective too. Simple actions such as removing garbage and domestic use containers can reduce over 90% of larval abundance and putting in window screens and closing doors can contribute to excluding over 80% of mosquito adults from homes [13]. Social mobilisation and communication programmes including modern channels should be developed with all national and local partners and community leaders. The integration of awareness-raising activities on the prevention and control of mosquito-borne diseases should be encouraged in church, school, and university programmes to educate church followers and students and use them as multipliers.

Moreover, our findings are very interesting, especially for local health authorities, epidemiologists, and other involved stakeholders; significant inferences can assist to accommodate the prevention strategies of MBVDs. The interpretation of the results concerning the perception is subject to certain limitations. The study design and the declarative nature of the data did not allow us to have absolute confidence in the different cognitive and behavioural statements. The high attitude score in this study could be explained by the fact that only the perceived risk and the perception of the responsibility of participants in the individual and collective prevention and control of mosquitoes were considered in the scoring of attitude. The importance of the perception of risk lies in its ability to determine our emotional, behavioural, and social reactions. Observational longitudinal studies would help to better understand the dynamics of the perceptions and practices of the population. Since KAP studies are more likely to be descriptive in nature, they often do not provide an in-depth insight into the reasons underlying the results. A complementary qualitative approach to our survey is therefore essential.

In the current study, the association between the qualitative binary variables of interest was assessed by using contingency analysis with a significance level of 0.05, as this p-value is commonly used to identify statistically significant associations. It could also increase the family-wise Type I error rate.

## 5. Conclusions

The findings of this study indicate that the population of Kinshasa lives in an environment conducive to the proliferation of mosquitoes and the spread of mosquito-borne diseases. However, the overall community knowledge regarding MBVD was poor in terms of mosquito biology, prevention, and control. Therefore, there is an urgent need to introduce multiple education programmes to raise their awareness and improve their knowledge. Particular emphasis should be placed on environmental sanitation, as it is essential to encourage this population to invest themselves in the hygiene of their living environment since it is also within their reach.

## Figures and Tables

**Table 1:** Socio-demographic characteristics of the participants.

Variable	No. of Respondents	Percent
Age Group		
18–33 years	702	47.9
34–70 years	762	52.5
Sex		
Male	575	39.3
Female	889	60.7
District		
Tshangu	509	34.8
Mont-Amba	388	26.5
Funa	207	14.1
Lukunga	360	24.6
Marital status		
Unmarried	737	50.3
Married	727	49.7
Education level		
Low education level	831	56.7
High education level	633	43.2
Occupation		
Medical personnel or student	303	20.7
Other	1161	79.3
Religion		
Christian	1234	84.3
Other	230	15.7

**Table 2:** Characteristics of participating households and their immediate environments.

Variable	No. of Respondents	Percent
Household size		
≤5	632	43.1
6–10	706	48.2
>10	126	8.6
Presence of children under 5 years old in the household	772	52.7
Homeownership		
Tenant	764	52.2
Owner	700	47.8
Source of water supply		
Tap water on the home premises	1024	69.9
Tap water away from the home premises	382	26.0
Well on the home premises	17	1.2
Well away from the home premises	49	3.3
Types of house walls		
Cement brick	1385	94.6
Sheet metal	53	3.6
Straw, clay, timber (wood)	26	1.8
Types of house roof		
Sheet metal	1370	93.6
Straw	94	6.4
Presence of net (insect screens) on windows	533	36.4
Description of house’s immediate surroundings		
Vegetation	567	38.7
Stagnant water collection	368	25.1
Storage water unit set outdoors	490	33.4
Any potential artificial or natural water container outdoors	330	22.5
An opened garbage can	897	61.2
Domestic animal keeping (rearing)	459	31.3

**Table 3:** Knowledge related to mosquito biology, vector role, and preventive measures.

Variable	No. of Respondents	Percent
Breeding places for mosquitoes		
Drain and stagnant polluted water	1178	80.3
Garbage	526	35.9
Unsafe waste disposal compost pit	137	9.3
Pits, drainage open underground soakage pits	141	9.6
Clean water collection	66	4.5
Ditches, ponds	148	10.1
Water storage tanks	80	5.4
Small containers	26	1.7
Storage and other water storage jars	24	1.6
Vehicle tyres	72	4.9
Coconut shells and broken utensils	35	2.3
Cracks in walls, tree holes	63	4.3
I don’t know	62	4.2
Other	28	1.9
Mosquito biting times		
Daytime (morning, afternoon)	63	4.3
Sundown	454	31.0
Night	571	39.0
Anytime	447	30.5
I don’t know	36	2.4
Season of the year mosquitoes are the most frequent		
Rainy season	704	48.0
Dry season	354	24.1
Both seasons	350	23.9
I don’t know	56	3.8
Can mosquitoes transmit disease to animals?		
Yes	288	19.7
No	1134	77.4
I don’t know	42	2.9
Can mosquitoes spread disease between animals and humans		
Yes	348	23.7
No	1090	74.5
I don’t know or don’t believe	26	1.8
Preventive measures		
Keep the environment clean, remove garbage or any uncovered container	1090	74.4
Use mosquito bed net	601	41.0
Keep cover over water source/storage unit container	151	10.3
Remove standing water/stagnant water	363	24.8
Spray insecticide	326	22.2
Fumigation	102	6.2
Use repellent	50	3.4
Use fan	67	4.5
Put mosquito screen (net) on house windows	130	8.8
Wear long clothes	25	1.7
I don’t know	33	2.2
Other (gasoline oil, detergent, etc.)	22	1.8

**Table 4:** Awareness of MBVDs and their transmission to humans and/or animals by mosquitoes.

	The Disease Can Be Transmitted by a Mosquito (*n* = 1464)	Being Aware of MBVD before the Survey (*n* = 1464)	MBVD That Can Be Transmitted between Humans and Animals (*n* = 348)
	***n* (%)**	***n* (%)**	***n* (%)**
Malaria	1423 (97.2)		119 (34.2)
Yellow fever	179 (12.2)	1269 (86.6)	9 (2.5)
Chikungunya	79 (5.4)	204 (13.9)	3 (0.8)
Zika	27 (1.8)	111 (7.5)	0.4
Dengue	22 (1.5)	55 (3.7)	
Rift Valley fever	9 (0.6)	26 (1.7)	3 (0.8)
West Nile fever		11 (0.7)	
O’nyong’nyong	2 (0.1)	8 (0.5)	3 (0.8)
Arbovirus	14 (0.9)		
Filariasis	1 (0.07)		3 (0.8)
Trypanosomiasis	17 (1.2)		29 (8.3)
Typhoid fever	69 (4.7)		11 (3.1)
Ebola	28 (1.9)		20 (5.7)
HIV	17 (1.1)		3 (0.8)
Rabies			16 (4.6)
Others	49 (3.4)		8 (2.2)
I don’t know	22 (1.5)		136 (39.0)

**Table 5:** KAP score about mosquitoes and mosquito-borne viral diseases (MBVDs) in Kinshasa, 2019.

Variable	Effective	Percent	IC 95%
Score of knowledge about breeding site			
Low	1392	95.1	93.8–96.1
High	72	4.9	3.8–6.1
Score of knowledge about mosquitoes’ period of activity			
Low	1018	69.5	67.0–71.8
High	446	30.5	28.1–32.9
Score of knowledge about the role of mosquitoes in spreading zoonoses			
Low	977	66.7	64.2–69.1
High	487	33.3	30.6–35.7
Score of knowledge about vector role of mosquitoes in arbovirus transmission			
Low	1208	82.5	80.4–84.4
High	256	17.5	15.5–19.5
Score of knowledge about arbovirosis			
Low	1407	96.1	94.9–97.0
High	57	3.9	2.9–5.0
Score of knowledge about mosquito control and prevention			
Low	1358	92.8	91.2–94.0
High	106	7.2	5.9–8.7
Overall score of knowledge			
Low	1346	92.0	90.4–93.2
High	118	8.0	6.7–9.6
Overall score of attitude			
Low	298	20.3	36.9–42.0
High	1166	79.7	57.9–63.3
Overall score of practice			
Low	1255	85.7	83.8–87.4
High	209	14.3	12.5–16.2

**Table 6:** Factors associated with a high level of knowledge about mosquitoes and MBVD in Kinshasa, 2019.

Variable	OR	X^2^	IC 95%	*p*
Factors associated with a high score of knowledge about breeding site				
Age group of 34–70 years	2.4	11.5	1.4–4.2	0.0002
Female sex	0.3	14.1	0.2–0.6	0.0001
Married	2.2	9.6	1.3–3.7	0.0016
Post-secondary educational level	2.0	7.6	1.2–3.2	0.002
Medical personnel or student	0.8	0.003	0.4–1.5	0.5
Non-Christian	0.9	0.003	0.5–1.8	0.5
Factors associated with a high score of knowledge about mosquitoes’ period of activity				
Age group of 34–70.years	1.3	7.6	1.1–1.7	0.002
Female sex	0.8	3.1	0.6–1.0	0.03
Married	1.0	0.1	0.8–1.3	0.3
Post-secondary education level	0.00	0.8	0.8–1.2	0.4
Medical personnel or student	1.0	0.15	0.7–1.2	0.3
Non-Christian	1.3	0.003	0.5–1.8	0.003
Factors associated with a high score of knowledge about role of mosquitoes in spreading zoonosis				
Age group of 34–70.years	0.9	0.0	0.7–1.2	0.5
Female sex	0.8	2.6	0.6–1.0	0.05
Married	0.9	1.1	0.7–1.0	0.1
Post-secondary education level	1.0	0.6	0.8–1.3	0.4
Medical personnel or student	0.9	0.3	0.6–1.1	0.2
Non-Christian	1.1	0.5	0.8–1.5	0.2
Factors associated with a high score of knowledge about vectors and the role of mosquitoes in arbovirus transmission				
Group age of 34–70.years	1.0	0.3	0.8–1.4	0.2
Female sex	0.8	1.9	0.6–1.1	0.08
Married	1.0	0.2	0.8–1.4	0.4
Post-secondary education level	0.9	0.3	0.6–1.2	0.2
Medical personnel or student	1.2	1.2	0.8–1.6	0.1
Non-Christian	1.3	2.4	0.9–1.9	0.06
Factors associated with a high score of knowledge about arboviruses				
Group age of 34–70.years	0.8	0.09	0.5–1.5	0.3
Female sex	0.6	2.0	0.3–1.1	0.07
Married	0.7	1.0	0.4–1.2	0.1
Post-secondary education level	1.1	0.2	0.6–2.0	1.2
Medical personnel or student	1.0	0.0	0.5–1.9	0.5
Non-Christian	1.7	2.8	0.9–3.3	0.05
Factors associated with a high score of knowledge about mosquito control and prevention				
Group age of 34–70.years	0.9	0.00	0.6–1.4	0.5
Female sex	1.1	2.0	0.7–1.6	0.3
Married	0.7	1.0	0.5–1.2	0.1
Post-secondary education level	1.2	0.9	0.8–1.8	0.1
Medical personnel or student	0.8	0.4	0.5–1.3	0.2
Non-Christian	1.1	0.05	0.6–1.8	0.3
Factors associated with a high global score of knowledge				
Group age of 34–70.years	1.1	0.16	0.7–1.6	0.3
Female sex	1.1	0.3	0.7–2.0	0.2
Married	1.2	1.3	0.8–1.8	0.1
Post-secondary education level	1.0	0.08	0.7–1.5	0.3
Medical personnel or student	0.8	0.4	0.5–1.3	0.2
Non-Christian	1.2	0.6	0.7–1.6	0.2

**Table 7:** Attitudes related to mosquito and mosquito-borne viral diseases.

Variable	No. of Respondents	Percent
Main source of the information		
Health professional/hospital	529	40.2
Family	344	26.1
Radio/television	333	25.3
School, college, university	233	17.7
Neighbours	117	8.9
Community leaders and volunteers	100	7.6
Megaphone public or government announcements	74	5.0
Internet, newspapers, SMS	74	5.0
Church/mosque	15	1.2
Other (traditional healer)	25	1.9
Impact of mosquitoes on daily life		
Health risk	1061	72.5
Nuisance	380	25.9
No concern	7	0.4
I don’t know	30	2.0
Other (disease, malaria, death)	103	7.0
In which locations are you often bitten?		
Indoors	741	50.6
Outdoors while I am at home	890	60.7
At workplace indoors	14	0.9
Outdoors while at workplace, recreational place	119	8.1
Everywhere	62	4.2
Nowhere	24	1.6
How often do you get bitten?		
Rarely	343	23.4
Sometimes	468	31.9
Regularly	653	44.6
During which time of the day are you often bitten?		
Daytime (morning, afternoon)	102	7.0
Sundown	528	36.0
Night	778	53.8
Anytime	177	12.0
Activity in your community leading to mosquito abundance		
Agriculture	206	14.0
Animal rearing	113	7.7
House building, road construction	157	11.6
Drainage and all blocked draining water channels	310	21.1
Garbage	260	17.7
Mechanic or automobile garage	12	0.8
Church services/prayers	14	0.9
Witchcraft/sorcery	14	0.9
Absence of sewage water draining system	29	1.9
Erosion, flooding, proximity to the river	15	1.1
Market, high population density	5	0.3
None	279	19
I don’t know	223	15.2

**Table 8:** Awareness about responsibility in the control and prevention of mosquitoes and mosquito-borne diseases.

	Self-Protection and Household	Community
	*n* (%)	*n* (%)
Individual responsibility	1068 (72.9)	546 (37.3)
Household head	128 (8.7)	114 (7.7)
Family members	40 (2.7)	7 (0.5)
Local community population	17 (1.2)	62 (4.2)
Health authorities	223 (15.2)	326 (22.2)
Local government administration	24 (1.6)	50 (3.4)
National government	173 (11.8)	245 (11.8)
Both government and population		96 (6.6)
God	8 (0.5)	2 (0.1)
None one	18 (1.2)	153 (10.4)
I don’t know		84 (5.7)

**Table 9:** Characteristics of participants associated with appropriate attitudes towards mosquitoes and mosquito-borne diseases (MBDs) in Kinshasa, 2019.

Variable	OR	X^2^	IC 95%	*p*
**Factors associated with appropriate attitude towards MBDs**				
Age group of 34–70 years	0.8	3.5	0.6–1.0	0.02
Female sex	0.9	0.3	0.7–1.1	0.2
Married	1.0	0.006	0.8–1.2	0.4
Post-secondary education level	1.0	0.1	0.8–1.2	0.3
Medical personnel or student	1.4	7.7	1.1–1.9	0.002
Non-Christian	0.8	0.9	0.6–1.1	0.1
High score of knowledge	1.2	0.9	0.8–1.8	0.1

**Table 10:** Practices related to mosquitoes and mosquito-borne diseases.

Variable	No. of Respondents	Percent
Measures were undertaken to reduce mosquito abundance on the property		
Put a cover over the water source/drinking water/storage unit/container	153	10.4
Empty flower pots/vases regularly	160	10.9
Cleaning environment	858	58.6
Empty other water containers serving as garbage collection	363	24.8
Fumigating	95	6.5
Remove garbage	166	11.3
Use of insecticides	380	25.9
Remove standing/stagnant water	239	16.3
Nothing	42	2.9
Use bed net	68	4.6
Close the house door	6	0.4
Measures that were undertaken to reduce or avoid mosquito bites		
Put mosquito screen on house windows	197	13.4
Sleep under bed net during the day	138	9.4
Sleep under bed net during the night	1158	79.1
Use of mosquito repellent during the day	19	1.3
Use of mosquito repellent during the night	44	3.0
Stay indoors	34	2.3
Use of fans	153	10.4
Fumigate and spray the home	232	15.8
Pray to God	15	1.0
Nothing	48	3.2
Wear long clothes	5	0.3
Other	48	3.2
Households having at least a mosquito bed net	1280	87.4
Slept under mosquito bed net last night	982	67.0
Source of mosquito bed net supply		
Mass distribution campaign	873	68.8
Shop/market	239	18.8
Health facilities	191	15.0
Other	26	2.0
Mosquito bed net with a hole in it	538	43.4
Any challenges in implementing preventive measures		
Yes	474	32.3
No	990	67.7
Types of challenges		
Have no time to apply these preventive measures	72	15
Lack of money and resources	204	42.9
Limited access to necessary items	92	19.3
Not a priority for me	34	7.1
I don’t believe these preventive measures are effective	61	12.8
Risk is low	15	2.9
Other	13	2.7

**Table 11:** Factors associated with good practice towards mosquitoes and mosquito-borne viral diseases (MBVDs) in Kinshasa.

Variable	OR	X^2^	IC 95%	*p*
Factors associated with appropriate attitude towards MBD				
Age group of 34–70.years	1.0	0.01	0.7–1.3	0.4
Female sex	1.2	2.1	0.9–1.7	0.07
Married	1.0	0.00	0.7–1.3	0.4
Post-secondary education level	1.0	0.02	0.7–1.3	0.4
Medical personnel and student	0.8	0.2	0.6–1.2	0.3
Non-Christian	0.5	6.4	0.3–0.8	0.003
High score in knowledge	1.4	1.6	0.8–2.3	0.1
Appropriate attitude	1.2	1.5	0.9–1.6	0.1

## Data Availability

All data supporting the study findings are included in this published article.

## Supplementary Materials

The following supporting information can be downloaded at: https://www.mdpi.com/article/10.3390/epidemiologia4010001/s1, File S1: Survey questionnaire; File S2: The scoring detail procedures for each KAP component.

## Abbreviations

MBVD	Mosquito-borne viral disease
YFV	Yellow fever virus
CHIKV	Chikungunya virus
DENV	Dengue virus
ZIKV	Zika virus
RVFV	Rift Valley fever virus
WNV	West Nile virus
ONNV	O’nyong’nyong virus
EVD	Ebola virus disease
KAP	Knowledge, attitude, and practice
